# Vaseline

**Published:** 1908-02-15

**Authors:** W. G. Hamm

**Affiliations:** Chillicothe, Ohio


### VASELINE.

BY W. G. HAMM, D. D. S., CHILLICOTHE, OHIO.

This is not meant to be a formal paper or exhaustive discussion of the use or value of vaseline for dental thero-peutic or mechanical purposes, but just to cite a few valuable uses of it in my hands. I have, after trying many other substances for lubricating carborundum wheels for cutting down stumps of teeth for crowning, etc., and concluded that wheels kept greased with vaseline will cut better, and with less pain to the patient than with any other lubricant. The vaseline also keeps the debris in a mass, adhering to the tooth or gums instead of allowing it to be scattered all over the mouth, or into the operator's and patient's eyes.

I have also found it of great value to keep burs from clogging with tooth debris when reaming out root canals or grinding out soft and sticky decayed dentine. If very thin twill tape be moistened with liquid vaseline, it will hold pumice very satisfactorily for polishing the proximal sur-faces of the teeth; it leaves the teeth smoother, and rubs the oil into the surface so that deposits do not reform so readily, and the teeth thus polished are not really so sensitive as they otherwise would be. It may be possible that vaseline and pumice do not remove stains so readily as pumice mixed with phenol sodique, which is a better solvent. Vaseline your pol shing brushes, and you will see how much better they will retain the polishing powders. Slightly grease the rubber dam with vaseline, and see how easily it will slip over the teeth and between those that are in very close contact. The liquid vaseline, called glymol, makes a splendid engine oil, and if flavored with a little perfume can be used as a lip ointment when taking impressions with plaster, or for oiling finishing strips and paper finishing disks. A felt pad saturated with vaseline is nice to slightly grease instruments used to adapt gutta percha and cement fillings. Small bottles containing a wad of cotton saturated with liquid vaseline and lysol, serve as excellent retainers for Kerr nerve drills and engine Gates-Gliddon drills. Vaseline is a good all-around friend. Keep it always near you.

